# Multi-level comparisons of cloacal, skin, feather and nest-associated microbiota suggest considerable influence of horizontal acquisition on the microbiota assembly of sympatric woodlarks and skylarks

**DOI:** 10.1186/s40168-017-0371-6

**Published:** 2017-12-01

**Authors:** H. Pieter J. van Veelen, Joana Falcao Salles, B. Irene Tieleman

**Affiliations:** Groningen Institute for Evolutionary Life Sciences, University of Groningen, P.O. box 11103, 9700 CC Groningen, The Netherlands

**Keywords:** Avian microbiota, Host-microbiome interactions, Horizontal acquisition, Phylogenetic clustering, Microbiome assembly

## Abstract

**Background:**

Working toward a general framework to understand the role of microbiota in animal biology requires the characterisation of animal-associated microbial communities and identification of the evolutionary and ecological factors shaping their variation. In this study, we described the microbiota in the cloaca, brood patch skin and feathers of two species of birds and the microbial communities in their nest environment. We compared patterns of resemblance between these microbial communities at different levels of biological organisation (species, individual, body part) and investigated the phylogenetic structure to deduce potential microbial community assembly processes.

**Results:**

Using 16S rRNA gene amplicon data of woodlarks (*Lullula arborea*) and skylarks (*Alauda arvensis*), we demonstrated that bird- and nest-associated microbiota showed substantial OTU co-occurrences and shared dominant taxonomic groups, despite variation in OTU richness, diversity and composition. Comparing host species, we uncovered that sympatric woodlarks and skylarks harboured similar microbiota, dominated by Proteobacteria, Firmicutes, Actinobacteria, Bacteroidetes and Acidobacteria. Yet, compared with the nest microbiota that showed little variation, each species’ bird-associated microbiota displayed substantial variation. The latter could be partly (~ 20%) explained by significant inter-individual differences. The various communities of the bird’s body (cloaca, brood patch skin and feathers) appeared connected with each other and with the nest microbiota (nest lining material and surface soil). Communities were more similar when the contact between niches was frequent or intense. Finally, bird microbiota showed significant phylogenetic clustering at the tips, but not at deeper branches of the phylogeny.

**Conclusions:**

Our interspecific comparison suggested that the environment is more important than phylogeny in shaping the bird-associated microbiotas. In addition, variation among individuals and among body parts suggested that intrinsic or behavioural differences among females and spatial heterogeneity among territories contributed to the microbiome variation of larks. Modest but significant phylogenetic clustering of cloacal, skin and feather microbiotas suggested weak habitat filtering in these niches. We propose that lark microbiota may be primarily, but not exclusively, shaped by horizontal acquisition from the regional bacterial pool at the breeding site. More generally, we hypothesise that the extent of ecological niche-sharing by avian (or other vertebrate) hosts may predict the convergence of their microbiota.

**Electronic supplementary material:**

The online version of this article (10.1186/s40168-017-0371-6) contains supplementary material, which is publicly available.

## Background

Symbiotic associations between animals and microorganisms are omnipresent and can play a fundamental role in animal evolution [1–4]. Working toward a general framework to understand the role of microbiota in animal biology requires the characterisation of animal-associated microbial communities and identification of the evolutionary and ecological factors shaping their variation. Although establishing a general theory for eco-evolutionary dynamics of animal-microbial interactions has recently received considerable attention [5–10], general conceptualisation has proven to be difficult. This challenge is hampered by fundamental gaps that need to be filled: first, the great variety of animal-microbiota systems encompasses diverse animal ecologies, reproductive modes and other life history traits that evolved in a wide range of (microbial) environments [11–18]. Second, host-microbial dynamics may vary among levels of biological organisation, e.g. between host species, among individuals and across the body. Third, animal hosts acquire their microbial symbionts vertically from parents and horizontally from the environment [19–21], but the strength of host-microbe associations and the relative contributions of vertical and horizontal acquisition are still unknown for most systems.

Current ideas on the strength of animal-microbe associations range from tight host-symbiont coevolution and interdependence to loose symbiotic interactions [1, 22–24]. In vertebrates, the relationships among host-associated communities and connections with environmental communities are understudied [25] despite their alleged role in horizontal acquisition [20]. Given the strong connection between symbiont transmission modes and the strength of host-microbe associations [4, 20], identifying the transmission modes in diverse systems and host lineages is a crucial step toward establishing general concepts. For instance, larger contributions of horizontal acquisition as compared to vertical symbiont transmission in microbiota assembly might reduce the strength of vertebrate-microbe associations, diminishing their adaptive potential and shaping their eco-evolutionary dynamics.

Integrative microbiota surveys appraising the nested nature of host biological organisation (that is, species, populations, individuals, organs, etc.) could provide improved insights in the relationships among microbial communities at various scales. Starting with variation at the host species-level, some studies argued that the phylogenetic inertia in gut microbiota variation among animals supports the idea that hosts and microbiota codiversified or coevolved [26–29]. However, host biogeography and behaviour can distinctly structure the bacterial microbiota of intraspecific populations, such as in humans [30] and great apes [15], and ecological factors such as diet [12, 31–34] and habitat features have been demonstrated to affect microbiota variation among individuals [35–39], populations [40] and species [28, 33, 41]. Moreover, the majority of vertebrate microbiome studies has focussed on gastrointestinal microbiota [12, 17, 29, 32], while many other components of vertebrate-microbiota systems (e.g. skin and oral microbiota) remained relatively underexplored in terms of origin, function, specificity, their reciprocal associations and their relationships with the microbial environment (but see [42]).

The phylogenetic relatedness within bird-associated microbiota could provide insights in the ecological or evolutionary processes that operate during microbiota assembly [43]. From a metacommunity perspective [44], microbial community assembly at different parts of the bird’s body (e.g., the cloaca, skin and feathers) can be viewed as discrete and permanent habitat patches harbouring local communities which are either neutrally assembled or are selected by local conditions and competition. Local communities are considered interconnected at a regional scale, which in this case comprises an individual’s body or territory, but could flexibly scale up to a population, a species, or a study site, depending on the question of interest [44]. Four key assembly processes are distinguished in the metacommunity framework: historical contingency, habitat filtering, dispersal-limitation and random assembly [44]. When applied to bird microbiota, historically contingent assembly predicts that bird species harbour and maintain distinctive microbiomes [26], retaining an ancestral signal in microbiome variation across the host phylogeny [6, 26]. Baas-Becking’s statement ‘everything is everywhere, but the environment selects’ [45] would predict habitat filtering in which local (a)biotic conditions select for particular microbial traits or members and thus predicts that microbiota vary among body sites (e.g. [13]). Dispersal-limited assembly would predict differences among body sites, and between body sites and the environment as a result of different dispersal probabilities, spatial segregation (i.e. contact frequency) or barriers to overcome [46]. Random (neutral) assembly predicts that local communities (body niches) are randomly assembled from the regional species pool (i.e. an individual microbiome or the bacteria present in a territory), which is expected to result in microbiota differences among individuals rather than that communities are mostly structured by body niche [44].

We aimed in this study to integrate different levels of comparison in a natural wild bird-microbiota system to evaluate the relationships among different animal-associated microbial communities and their association with environmental microbial communities in two sympatric bird species. We first described the bacterial communities of different body parts (cloaca, brood patch skin, feather) and nest environments (nest lining and surface soil) of sympatric woodlarks *Lullula arborea* and skylarks *Alauda arvensis* (Aves; Alaudidae). Then, to reveal patterns of resemblance at different levels of biological organisation of bird-associated microbiotas, we compared bacterial community diversity and composition between host species, among individual birds and among distinct body parts along with their nest environments. Finally, we investigated the phylogenetic structure of the microbiota at each body part and used the resulting patterns to speculate about potential assembly processes that contributed to shaping bird-associated microbiota in the wild.

## Methods

### Study site and species

We studied sympatric breeding lark species at Aekingerzand, the Netherlands (N 52°55′; E 6°18′; described in [47]). Woodlarks *Lullula arborea* and skylarks *Alauda arvensis* scrape shallow cups on bare soils to build their nests, primarily composed of dry grass stems and often adjoining heather or grass tussocks. Adult woodlark and skylark diet largely comprises arthropods during the breeding season (~ 70–80%), complemented by plant material and seeds [48, 49]. Breeding territories of both larks overlap, but in contrast to skylarks, woodlarks also exploit the area’s peripheral forest clearings.

### Sample collection

Between March and July 2014, we sampled adult female woodlarks (*n* = 15) and skylarks (*n* = 14) and their nest locations, comprising cloacal, brood patch skin, body feather, nest lining material and surface soil samples. We collected a total of 120 samples, including 20 complete sets and incomplete sets for nine females (see details in Table S1 [Additional file 1] or the metadata [MG-RAST project ID 21350]). We handled and sampled birds exclusively with 70% ethanol-sterilised gloves and equipment. We sampled the external and internal microbial niches of the bird in three ways: first, we swabbed the bare skin of the brood patch with a sterile cotton swab moistened with sterilised PBS solution. We then inserted a sterile cotton swab through the cloaca and sampled the microbiota by gentle rotation. Finally, we clipped the distal half of ~ 5 brood patch-lining body feathers with scissors and tweezers. After we released the bird, we collected nest lining material (~ 3 grass stems) from the centre of the nest cup and collected a composite soil sample of the surface within a 50-cm radius facing the nest entrance. All samples were stored in sterilised 2-ml screw-cap vials that we kept on ice in the field (< 12 h post-collection) and then stored at − 20 °C.

### DNA extraction and 16S rRNA gene amplification and sequencing

We aseptically peeled cloacal and brood patch skin swabs from the stalks to loosen cotton fibres and added all cotton to extraction tubes. We further transferred ~ 5 brood patch-lining feathers and ~ 3 stems of nest lining material each into sterile 15-ml tubes and added 978 μl sodium phosphate buffer with 122 μl MT buffer (kit reagents of FastDNA™ SPIN Kit for Soil; MP Biomedicals, Santa Ana, CA, USA). We then vortexed the tubes for 10 s using a Vortex-Genie2 (MoBio Laboratories Inc., Carlsbad, CA), sonicated the tubes for 15 min, and vortexed for another 10 min to detach bacterial cells from the source materials. We transferred the cell suspensions to lysis tubes to complete the extraction. On average, we used (± SEM) 0.3 ± 0.01 g soil per surface soil sample for total DNA extraction. We followed the manufacturer’s protocol for all samples, but we achieved enhanced cell lysis by bead beating for 1 min three times on a mini bead beater (BioSpec Products, Bartsville, OK, USA). We eluted DNA in 100 μl PCR-grade water (Ambion, Austin, TX, USA) and subsequently quantified DNA concentrations using the Quant-it PicoGreen dsDNA kit (Molecular Probes, Invitrogen, Eugene, OR). We then amplified the V4/V5 region of the 16S rRNA gene using the primers 515F and 926R on the following thermal cycling protocol: 5 min at 95 °C, 35 cycles with 40 s at 95 °C, 45 s at 56 °C, 40 s at 72 °C and finally 10 min at 72 °C. Nine collected samples did not amplify during PCR and could not be included for downstream analysis; see Supplementary Table S1 (Additional file 1) or the metadata file (Additional file 2) for details. Finally, at GenoToul (INRA, Toulouse, France), purified amplicons (QIAquick gel extraction Kit, QIAGEN GmbH, Hilden, Germany) were extended with Illumina adapters using PCR, and 7 pM of equal amounts of PCR products including adapters was then sequenced using the 2 × 250 bp v2 chemistry on an Illumina MiSeq platform.

### Sequence data processing

We processed raw 16S rRNA gene sequence data using QIIME 1.9.0 [50]. Sequence reads were demultiplexed and quality filtered at Genotoul using the default settings in QIIME: quality score ≥ 25, maximal ambiguous base calls = 6, maximum length of homopolymer run = 6, no primer mismatches. We then joined paired-end reads and truncated reverse primers from joined reads. Subsequently, we commenced an open-reference OTU-picking strategy against the Greengenes reference database (v. 13.8) [51] at 97% identity using the *uclust* algorithm [52], and de novo OTU picking of a 0.1% random subset of reads that failed to match the reference set, following the QIIME tutorial [53]. Subsequently, we picked representative sequences for all OTUs prior to merging both OTU tables. We removed all singletons to reduce the effects of sequencing errors on alpha diversity estimates. We then annotated taxonomic information against Greengenes (v. 13.8, 97% identity reference set) and subsequently aligned representative sequences using default settings with PyNast [54]. We identified and removed chimeric sequences using the *uchime* algorithm in the *usearch81* toolkit [55] and constructed a phylogenetic tree using FastTree [56]. Finally, we filtered OTUs assigned to Archaea, Chloroplast and Mitochondria from the OTU table and retained OTUs with abundances > 0.01% of the total abundance. Rarefaction curves showed that OTU richness had not reached saturation, where Shannon diversity had levelled at 5000 reads per sample for each sample type (Figure S1 [Additional file 1]). Despite moderate coverage and saturation in our data set, the estimated total diversity clearly differed among sample types (Chao1; Figure S1c [Additional file 1]) and Shannon diversity estimates are likely to be unaffected at ~ 5000 reads per sample. We removed a single low coverage sample (i.e. brood patch skin sample with 1049 reads) and subsequently rarefied all samples to 5000 reads/sample prior to analyses. The QIIME script of our pipeline is available in Additional file 3 and its associated data files in Additional files 2, 4, 5 and 6.

### Statistical analyses

We analysed bacterial diversity based on rarefied and unrarefied data using the R packages *phyloseq* (v. 1.14.0; [57]) and *vegan* (v. 2.4-0; [58]) using R statistical software (v. 3.2.3; [59]). All R scripts are accessible in Additional file 7.

### Diversity within bacterial communities

We calculated OTU richness and Shannon's diversity index (hereafter 'Shannon diversity') from rarefied data and used ANOVA with Tukey-Kramer post hoc tests to analyse group differences between lark species (‘Woodlark’, ‘Skylark’) and sample types (‘Cloaca’, ‘Brood patch skin’, ‘Feather’, ‘Nest lining’, ‘Surface soil’), with verification of the normality of residual errors (Q-Q plots) and homoscedasticity (fitted values ~ residuals plot). We report adjusted *P*-values for pairwise Tukey-Kramer contrasts using the default single-step method of the *multcomp* package [60]. We evaluated general differences in OTU richness and Shannon diversity among individual females and their nests by modelling Nest ID as a random factor to our initial ANOVA, fitted by restricted maximum likelihood (REML) using the *nlme* package [58]. These models were run on all sample types and on subsets containing either female-associated or nest-associated samples. We tested the significance of the random Nest ID effect using a likelihood ratio test comparing the REML-fitted mixed-effects model with a REML-fitted linear model without the random Nest ID term [61] and calculated the explained variance proportion by the random term using the *MuMIn* package [62]. We compared and visualised OTU co-occurrences among the different sample types using venn diagrams for woodlarks and skylarks separately using the methods provided by the Hallam Lab [https://github.com/hallamlab/mp_tutorial/wiki/Introduction-to-Downstream-Analysis-in-R]. For both host species separately, we identified and described the most abundant OTUs in each bird-associated sample type with a mean abundance threshold of 5% across all samples per sample type using the *core_microbiome* function provided by David Elliott [https://github.com/davidelliott/core-microbiome/blob/master/core-microbiome.Rmd].

In order to identify differential OTU abundance in woodlarks and skylarks for each sample type, we performed analysis of composition of microbiomes (ANCOM) [63] with a critical false discovery rate (FDR)-corrected *q*-value of 0.05.

### Pairwise community similarity between hosts and among sample types

We assessed bacterial community composition (beta diversity) using weighted UniFrac [64] and Bray-Curtis dissimilarities to evaluate phylogenetic similarity among groups. We performed principal coordinate ordination analysis (PCoA) using *vegan*. In order to test if different sample types and lark species affected community clustering and group dispersion (i.e. mean distance to the cluster centroid to represent the variation among individuals within a sample type), we modelled weighted UniFrac distances and Bray-Curtis dissimilarities from an OTU-level table using PerMANOVA with 999 permutations (‘adonis’ function in *vegan*) [65, 66]. We used the ‘betadisper’ function [67] in *vegan* to evaluate the degree of within-group dispersions among sample types and calculated group differences using Kruskal-Wallis tests with a post hoc Dunn’s test for multiple comparisons [68]. We reported FDR-corrected *q*-values. This method allowed us to determine whether PCoA clustering of weighted UniFrac distances were due to location effects or dispersion effects.

### Among-individual and among-sample type effects on pairwise similarities

In order to test whether overall differences among females could explain additional variation in community clustering, i.e. individuality of female-nest dyads, we added Female ID as a predictor to the PerMANOVA, with permutations restricted by lark species. We calculated the phylogenetic distance (weighted UniFrac) among pairs of sample types and used ANOVA to determine mean pairwise differences among sample types. All effect sizes and their significance were evaluated with post hoc pairwise Tukey-Kramer contrasts. We additionally evaluated intrinsic structure of the data using partioning around medoids (PAM) using the *cluster* package [69] to evaluate how samples would cluster without prior metadata information.

### Null model of phylogenetic community structure

We used a null modelling approach with our 97% identity-based community tables to evaluate the phylogenetic structure of OTUs within each sample type community (following [70, 71]). We used the *picante* package (v. 1.6-2; [72]) to calculate the average distance between co-occurring phylogenetic relatives (observed mean nearest taxon distance, MNTD_obs_) and the mean pairwise phylogenetic distance (observed mean phylogenetic distance, MPD_obs_) among all pairs of species in each sample (local community). Then, by comparing observed values with a null distribution (MNTD_null_ and MPD_null_) following [73], we calculated the standardised effect size for every sample and each metric, which is referred to as − 1 times nearest taxon index (NTI) or net relatedness index (NRI) [70, 71], respectively. We generated the null distributions using the ‘independent swap’ algorithm [74, 75], referred to as ‘null model 4’ in [71], in which species co-occurrences were randomised 1000 times per randomisation, maintaining species richness and occurrence frequencies in each sample type community’s phylogenetic tree. We finally inferred whether phylogenetic clustering or phylogenetic evenness was observed in each sample type (which is expected when the average NTI or NRI value is different from the null communities) by testing whether the mean NTI or NRI value of each sample type differed from zero. Tests were performed using ANOVA and post hoc Tukey-Kramer pairwise contrasts.

## Results

Our sequencing effort produced 5,054,382 quality filtered sequences after removal of singletons, clustered in 1148 OTUs with a minimum abundance of 0.01%. The coverage range was 5225–80,815 reads per sample in the analysed samples. The ranges per sample type were as follows: cloaca, 5225–55,146; brood patch skin, 8938–80,579; feathers, 8198–64,463; nest lining, 7511–64,091; and surface soil, 11,275–50,995. Rank-abundance plots for the five sample types were similar (Figure S2 [Additional file 1]), indicating dominance of a few types and a long tail of less abundant OTUs. Samples were rarefied to 5000 sequences to avoid biases due to sequencing effort. Of 1148 OTUs, 4.9% could be assigned to species level, 35.4% to genus level and 83.9% to family level. 

### Richness and diversity of bird- and nest-associated microbiota of woodlarks and skylarks

**Fig. 1:**
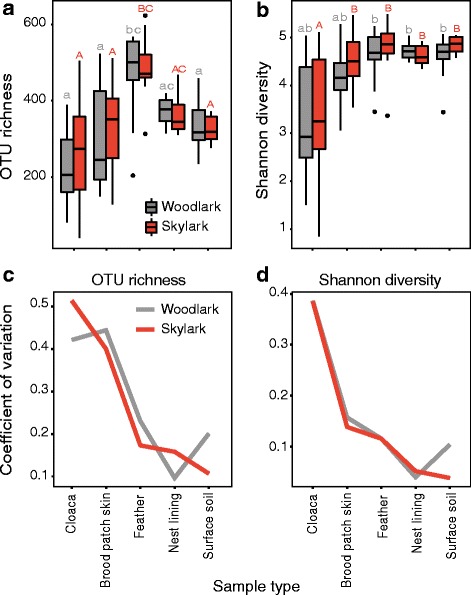
Alpha diversity metrics across sample types of sympatric woodlarks and skylarks. Bacterial OTU richness **a** and Shannon diversity **b** are consistently variable within sample types, and **c**, **d** unbiased estimates of the coefficients of variation show decreasing trends of variability of OTU richness and Shannon diversity of bacterial communities, evaluated for each sample type and ordered from the bird’s internal community outward to the surface soil communities. **a**, **b** Letters represent pairwise contrasts (P < 0.01) of sample type means of woodlarks (lower case grey) and skylarks (capital red)

Body feathers harboured the richest microbiota compared to cloaca, brood patch skin, nest lining and surface soil communities in both larks. In woodlarks, the mean (± SEM) number of OTUs recovered from feather communities (473 ± 30) was almost double, and in skylarks (478 ± 25) about 1.7 times the number found in their respective cloacal samples. OTU richness did not differ between lark species in any of the sample types (lark species: *F*_1,100_ = 0.17, *P =* 0.86; sample type × lark species: *F*_4,100_ = 0.28, *P* = 0.89), but OTU richness differed among sample types (*F*_4,104_ = 18.2, *P* < 0.001; Fig. 1a, Table 1). Shannon diversity varied among sample types (*F*_4,104_ = 16.65, *P* < 0.001), but not between the woodlarks and skylarks (*F*_1,104_ = 1.07, *P* = 0.30) (Fig. 1b). Mean (± SEM) Shannon diversity in cloacal communities of woodlarks (3.28 ± 0.36) and skylarks (3.39 ± 0.35) was lower than that in other sample types, though in woodlarks, the difference with brood patch skin communities received no statistical support (Table 1). An OTU table with all reads that did not match the Greengenes reference set produced similar patterns (Figure S3a, b; Additional file 1).

**Table 1:** Pairwise ANOVA statistics of bacterial alpha diversity among sample types

		OTU richness							
		Pairwise Tukey-Kramer contrasts among sample types							
		Woodlark				Skylark			
Pairwise comparison		Estimate	SE	*t*	*P*	Estimate	SE	*t*	*P*_adj_
Cloaca	Brood patch skin	− 73.75	40.854	− 1.806	0.728	− 54.44	40.087	− 1.358	0.936
	Feathers	− 242.83	39.171	− 6.199	< 0.001	− 212.08	40.087	− 5.290	< 0.001
	Nest lining	− 139.52	39.171	− 3.562	0.019	− 89.65	45.87	− 1.954	0.630
	Surface soil	− 106.15	41.897	− 2.534	0.262	− 51.74	42.431	− 1.219	0.967
Brood patch skin	Feathers	− 169.08	40.087	− 4.218	< 0.01	− 157.64	41.723	− 3.778	< 0.01
	Nest lining	− 65.78	40.087	1.641	0.824	− 35.21	47.310	− 0.744	0.999
	Surface soil	− 32.40	42.754	0.758	0.999	2.697	43.98	0.061	1.000
Feathers	Nest lining	103.31	38.380	2.692	0.190	122.43	47.310	2.588	0.237
	Surface soil	136.68	41.158	3.321	0.04	160.33	43.980	3.646	0.015
Nest lining	Surface soil	33.37	41.158	0.811	0.998	37.91	49.312	0.769	0.999
		Shannon diversity							
		Pairwise Tukey-Kramer contrasts among sample types							
		Woodlark				Skylark			
Pairwise comparison		Estimate	SE	*t*	*P*	Estimate	SE	*t*	*P*_adj_
Cloaca	Brood patch skin	− 0.87	0.286	− 3.061	0.095	− 1.14	0.280	− 4.069	< 0.001
	Feathers	− 1.37	0.274	− 5.012	< 0.001	− 1.41	0.280	− 5.041	< 0.001
	Nest lining	− 1.46	0.274	− 5.319	< 0.001	− 1.27	0.320	− 3.971	< 0.01
	Surface soil	− 1.32	0.293	− 4.491	< 0.001	− 1.48	0.267	− 5.009	< 0.001
Brood patch skin	Feathers	− 0.50	0.280	− 1.779	0.819	− 0.27	0.292	− 0.934	0.993
	Nest lining	− 0.58	0.280	− 2.079	0.626	− 0.13	0.331	− 0.403	1.000
	Surface soil	− 0.44	0.299	− 1.477	0.941	− 0.35	0.308	− 1.124	0.993
Feathers	Nest lining	− 0.08	0.268	− 0.313	1.000	0.14	0.331	0.421	1.000
	Surface soil	0.06	0.287	0.199	1.000	− 0.07	0.308	− 0.238	1.000
Nest lining	Surface soil	0.14	0.288	0.491	1.000	− 0.21	0.345	− 0.616	1.000

The degree of variation in OTU richness and Shannon diversity within each sample type was largest in the bird-associated sample types compared to the nest environment (Fig. 1c). In an attempt to explain the substantial variation in richness and diversity, we tested whether part of the variation might be due to general differences among females. A random Female ID term substantially improved model support (likelihood ratio test (LRT); χ^2^ = 6.51, *P* < 0.05) and explained 10% of the total variance of OTU richness, while sample type explained 41% of the variance within nests. Female ID did not significantly explain variation in Shannon diversity (LRT; χ^2^ = 0.55, *P* = 0.46). We also modelled the random Female ID term on OTU richness in a data set restricted to the bird-associated samples (i.e. excluding nest lining and surface soil) (LRT; χ^2^ = 5.17, *P* < 0.05) and found that the proportion of variance explained by Female ID increased to 18%, with sample type accounting for 43% of variance within females. A similar model that included only nest lining and surface soil communities did not reveal such individual differences (LRT; χ^2^ = 0.32, *P* = 0.57), thus demonstrating that Female ID primarily predicted richness variation in bird-associated bacterial communities, but not among nest-associated communities.

### OTU co-occurrence patterns between bird- and nest-associated microbiota of woodlarks and skylarks

**Fig. 2:**
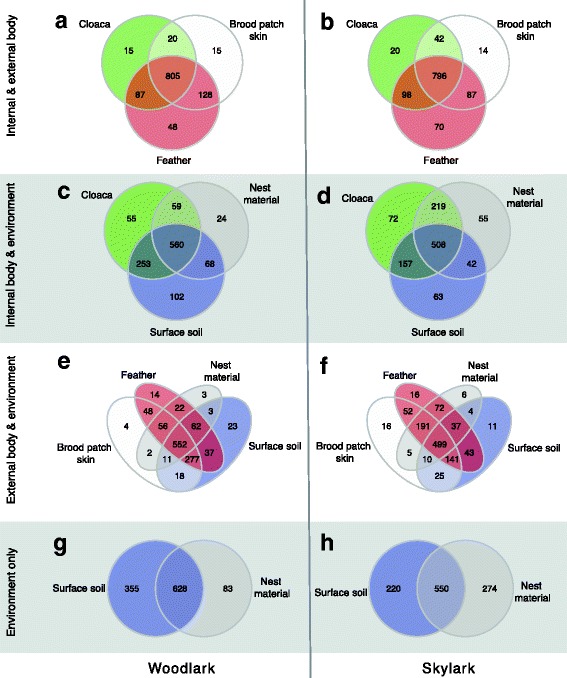
Venn diagrams of co-occurring OTUs among sample types for woodlarks and skylarks. **a**, **b** Comparison of internal (cloaca) and external (brood patch skin and feather) communities. **c**, **d** Internal and environmental (nest material and surface soil) communities. **e**, **f** External and environmental communities. **g**, **h** Environmental communities of woodlarks and skylarks. Numbers in each compartment denote the number of unique and shared OTUs of the (non-)overlapping communities

Analysis of OTU co-occurrence patterns showed that 78, 80 and 89% of the OTUs identified in cloacal, brood patch skin and feathers, respectively, were shared between woodlarks and skylarks. Comparisons of sample types revealed that the majority of OTUs on the female body were shared among cloacal, skin and feather communities and in similar proportions for both larks (woodlark, 72%; skylark, 71%; Fig. 2a, b). Cloacal microbiotas of woodlarks and skylarks harboured a small proportion of unique OTUs (woodlark, 5%; skylark, 7%) compared to communities of the nest environment with which they shared 50 and 46%, respectively (Fig. 2c, d). With a majority of OTUs shared by both external body niches (brood patch skin and body feathers) and both environmental communities (woodlark, 51%; skylark, 44%), skin and feathers harboured few unique OTUs (Fig. 2e, f). Our proxy for the microbial environment of breeding larks, i.e. nest material and surface soil around the nest, showed that nest materials and surface soils each harboured a substantial number of unique OTUs and illuminated the complexity of the microbial environment (Fig. 2g, h).

### Relative taxon abundances in the microbiota of woodlarks, skylarks and their nests

**Fig. 3:**
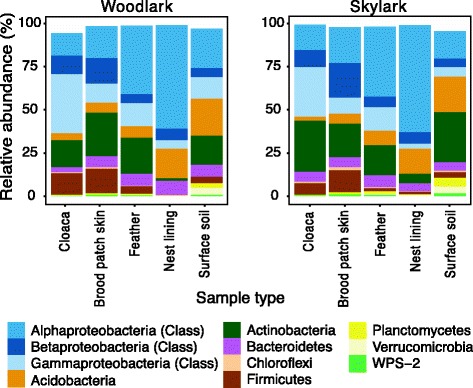
Barplots of relative abundances of the most abundant bacterial phyla and Proteobacteria classes in each sample type in woodlarks and skylarks. Calculations were based on rarefied data (5000 reads/sample)

Of a total of 15 identified bacterial phyla, 9 phyla dominated the lark’s microbiota (cumulative abundance > 94.3%) and included the bacterial phyla characterised as dominant in avian gut microbiota studies: Proteobacteria, Acidobacteria, Actinobacteria, Bacteroidetes and Firmicutes. The relative abundances of each of the 15 phyla significantly varied among the sample types (ANCOM, FDR *q* < 0.05), but the general pattern was consistent in both lark species (Fig. 3). Proteobacteria comprised the most dominant phylum in the bird-associated microbiota and on nest lining material, but were relatively less abundant in surface soil communities. However, within these Proteobacteria-dominated microbiota, class-level patterns showed that cloacal microbiota harboured on average the largest fraction of Alphaproteobacteria, brood patch skin microbiota the highest proportion of Betaproteobacteria and feathers and nest lining communities predominantly Gammaproteobacteria (Fig. 3). Actinobacteria comprised the second dominant phylum in bird microbiota and, in contrast to Proteobacteria, comprised a larger proportion of soil communities than of nest material communities. Acidobacteria were relatively more abundant in the environmental communities than in bird microbiota, and Firmicutes appeared relatively more abundant in cloacal and brood patch skin communities. The patterns were, however, highly variable among individuals and particularly at higher taxonomic resolution (Figure S4a–f [Additional file 1]). ANCOM identified only 4 out of 1148 OTUs (Firmicutes: Aerococcaceae OTU1110381, Proteobacteria; Neisseriaceae OTU965048, FBP; OTU224307, Planctomycetes; Gemmataceae OTU1042) that varied significantly in abundance between woodlarks and skylarks, presumably resulting from the large variation among individual birds. These higher-level abundance patterns (Fig. 3) were partly the result of a few dominating OTUs (Figure S4 [Additional file 1]). OTUs belonging to Oxalobacteraceae and Enterobacteriaceae were revealed as dominant Proteobacteria OTUs in cloacal communities of both lark species (Figure S4a, b [Additional file 1]). Furthermore, the most abundant Actinobacteria OTUs in most cloacal microbiota samples were represented by Intrasporangiaceae in woodlarks and Corynebacteriaceae in skylarks. In the brood patch skin communities of woodlarks and skylarks, OTUs belonging to the families Oxalobacteraceae, Enterobacteriaceae and Methylocystaceae were the most dominant Proteobacteria taxa, and a Pseudonocardiaceae OTU appeared the most abundant OTU within Actinobacteria in both larks, with Intrasporangiaceae also being a dominant taxon on woodlark skin (Figure S4c, d [Additional file 1]). An OTU belonging to Solibacteraceae dominantly represented the Acidobacteria, and an OTU belonging to Chitinophagaceae dominantly represented Bacteroidetes in both species. Feather microbiota constituted of the same dominant OTUs as brood patch skin microbiota but with an additional high prevalence of Acetobacteriaceae as a member of Proteobacteria and an OTU representing Acidobacteriaceae replacing the Solibacteraceae OTU in the Acidobacteria phylum (Figure S4e, f [Additional file 1]).

### Community resemblance between host species, among individuals and within each sample type

**Fig. 4:**
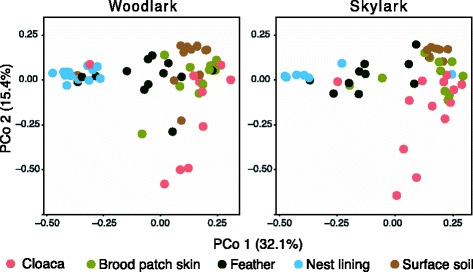
Beta diversity of bacterial communities associated with different sample types of sympatric lark species. Principal coordinate analysis (PCoA) of weighted UniFrac distances among sample types is shown along the first two principal coordinate axes and was calculated on a single rarefied data set and visualised for both species separately. Clustering significance was determined by PerMANOVA. Sample type (33%, *P* < 0.001) and lark species (1%, *P* < 0.05) explained total variation

Analysis of β-diversity based on weighted UniFrac distances revealed differential community clustering of sample types (PerMANOVA, pseudo-*F* = 13.2, df = 4, 104, *R* ^2^ = 0.33, *P* < 0.001; Fig. 3) but only weakly supported clustering of woodlarks and skylarks (pseudo-*F* = 2.19, df = 1, 104, *R* ^2^ = 0.01, *P* < 0.05). In addition to sample type (*R* ^2^ = 33%) and lark species (*R* ^2^ = 1%), Female ID explained an additional 20% of variation in clustering of weighted UniFrac distances (PerMANOVA, pseudo-*F* = 1.24, df = 27, 77, *R* ^2^ = 0. 20, *P* < 0.05), suggesting significant bacterial community convergence at the level of individual hosts. Consistent results based on Bray-Curtis are shown in Figure S5 (Additional file 1). Note that PCoA clustering of samples using a weighted UniFrac matrix based on an OTU table constructed with the full set of reads that did not match the Greengenes reference set were similar to the patterns presented here (Figure S3c [Additional file 1]). Sample cluster analysis by partitioning around medoids (PAM) did not reveal an optimal number of *K* clusters (Figure S6a [Additional file 1]), but showed that nest lining and surface soil communities clustered reasonably good when *K* = 5 (i.e. number of expected clusters; Fig. 4) was chosen for ordination (Figure S6b, c [Additional file 1]). Female-associated samples were only modestly clustered according to sample type when ordination was based on intrinsic structure of the data. As expected, samples did not cluster in PCoA by individual female since sample type was clearly the strongest driver in community clustering (Fig. 4).

### Comparing group dispersions in community composition among sample types

**Fig. 5:**
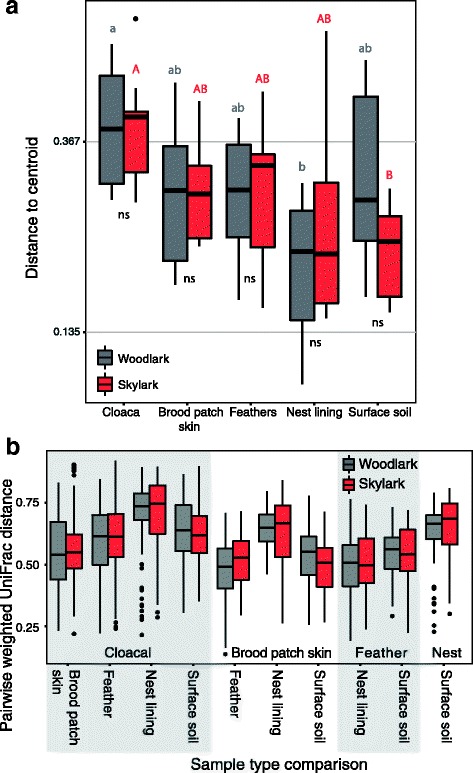
Group dispersion within and among microbial niches. Distances to the cluster centroids represent the variation among individuals within sample types and depicts how dispersion varies among host species and sample types. **a** Group dispersion in PCoA among individuals within each sample type. **b** Pairwise weighted UniFrac distances among sample types. Weighted UniFrac distances were calculated based on rarefied data (5000 reads/sample). **a** Letters denote Dunn’s contrasts (FDR *q* < 0.05) of median distances between pairs of sample types for woodlarks (lower case grey) and skylarks (capital red). Interspecific contrasts of mean distances are expressed below boxes of each sample type (ns = not significant). **b** Shaded/unshaded areas denote a base sample type, in which weighted UniFrac distance was pairwise compared with the associated sample types labelled along the *x*-axis. Statistics of between sample type comparisons are reported in Table 2

As a measure of inter-individual variation in PCoA clustering of weighted UniFrac distances, the median distances to the cluster centroid of sample types varied (*χ* ^2^ = 34.4, df = 9, *P* < 0.001). This measure of community variability, also referred to as group dispersion, was highest in cloacal communities (Fig. 5a), but this difference received only statistical support for the comparisons with the dispersion in nest lining communities (woodlark: Kruskal-Wallis *H* = 4.23, *P* < 0.001; skylark: *H* = 2.75, *P* < 0.05) and in surface soil (woodlark: *H* = 1.63, *P* = 0.09; skylark: *H* = 3.71, *P* < 0.01). Pairwise Dunn’s contrasts revealed that the distances to the cluster centroids of none of the sample type communities differed between woodlarks and skylarks (all pairwise comparisons: FDR *q* > 0.05; Fig. 5a). Since group dispersions differed only between cloacal communities and either nest lining or soil communities, the question remains whether the significance of PCoA clustering arose through location effects or dispersion effects. Because nest lining and surface soil communities each clustered very clearly (Fig. 4), and because the group dispersions among bird-associated sample types did not differ (Fig. 5a), significant sample clustering by sample type is likely a true location effect rather than an effect of dispersion. In addition, neither nest lining communities (PerMANOVA, pseudo-*F* = 1.43, df = 1, 18, *R* ^2^ = 0.07, *P* = 0.18) nor surface soil communities (PerMANOVA, pseudo-*F* = 1.57, df = 1, 17, *R* ^2^ = 0.08, *P* = 0.09) clustered separately for woodlarks and skylarks (Fig. 4).

### Comparing community resemblance among sample types

As a measure of phylogenetic similarity among the various sample types, mean pairwise weighted UniFrac distances among sample types varied substantially among all ten pairwise comparisons, but this variation was consistent for woodlarks and skylarks. Across among-sample type comparisons, community variability varied (*F*_9,2399_ = 78.66, *P* < 0.001; Fig. 5b). Higher mean weighted UniFrac between cloacal and nest lining communities suggests that, on average, these communities least resembled each other (Fig. 5b, Table 2). Cloacal communities were least similar to those on feathers and in soils. Instead, cloacal communities mostly resembled skin communities, skin communities were most similar to feather and soil communities and feather communities mostly resembled skin and nest lining communities. As measures of the nest environment, nest lining and soil communities were markedly different. These resemblance patterns imply that physical contact and spatial proximity among bird- and/or nest-associated bacterial niches influenced the degree of resemblance among them. Here, host species contributed to explaining similarity among sample types, which was demonstrated by a significant ‘comparison ID × lark species’ interaction (*F*_9,2399_ = 2.37, *P* < 0.05). The general patterns were similar for both host species, and post hoc pairwise contrasts indicated only small effects (see Table 2 for all pairwise statistics).

**Table 2:** ANOVA statistics of phylogenetic dispersion among sample types based on weighted UniFrac

				Woodlark			Skylark		
Comparison I		Comparison II		Estimate (SE)	*t* statistic	FDR *q*-value^a^	Estimate (SE)	*t* statistic	FDR *q*-value^a^
Cloaca	Brood patch skin	Cloaca	Feathers	− 0.05 (0.015)	− 3.69	**0.050**	− 0.04 (0.015)	− 2.99	0.35
Cloaca	Brood patch skin	Cloaca	Nest lining	− 0.17 (0.015)	− 11.62	**< 0.001**	− 0.14 (0.016)	− 8.36	**< 0.001**
Cloaca	Brood patch skin	Cloaca	Surface soil	− 0.09 (0.016)	− 6.08	**< 0.001**	− 0.06 (0.015)	− 4.07	**0.013**
Cloaca	Feathers	Cloaca	Nest lining	− 0.12 (0.014)	− 8.29	**< 0.001**	− 0.09 (0.016)	− 5.72	**< 0.001**
Cloaca	Feathers	Cloaca	Surface soil	− 0.04 (0.015)	− 2.73	0.57	− 0.02 (0.015)	− 1.23	1.00
Cloaca	Nest lining	Cloaca	Surface soil	0.07 (0.015)	5.00	**< 0.001**	0.08 (0.017)	4.39	**0.004**
Cloaca	Brood patch skin	Brood patch skin	Feathers	0.06 (0.015)	3.76	0.041	0.05 (0.015)	3.25	0.18
Cloaca	Brood patch skin	Brood patch skin	Nest lining	− 0.11 (0.015)	− 7.17	**< 0.001**	− 0.05 (0.017)	− 3.09	0.28
Cloaca	Brood patch skin	Brood patch skin	Surface soil	0.00 (0.016)	− 0.15	1.00	0.07 (0.016)	4.49	**0.003**
Cloaca	Feathers	Brood patch skin	Feathers	0.11 (0.014)	7.68	**< 0.001**	0.09 (0.015)	6.12	**< 0.001**
Cloaca	Feathers	Brood patch skin	Nest lining	− 0.05 (0.014)	− 3.71	**0.047**	− 0.01 (0.017)	− 0.59	1.00
Cloaca	Feathers	Brood patch skin	Surface soil	0.05 (0.015)	3.35	0.14	0.12 (0.016)	7.20	**< 0.001**
Cloaca	Nest lining	Brood patch skin	Feathers	0.22 (0.014)	15.79	**< 0.001**	0.19 (0.017)	10.98	**< 0.001**
Cloaca	Nest lining	Brood patch skin	Nest lining	0.06 (0.014)	4.40	**0.003**	0.08 (0.019)	4.42	**0.003**
Cloaca	Nest lining	Brood patch skin	Surface soil	0.17 (0.015)	10.89	**< 0.001**	0.21 (0.018)	11.77	**< 0.001**
Cloaca	Surface soil	Brood patch skin	Feathers	0.15 (0.015)	9.86	**< 0.001**	0.11 (0.016)	7.01	**< 0.001**
Cloaca	Surface soil	Brood patch skin	Nest lining	− 0.01 (0.015)	− 0.79	1.00	0.01 (0.018)	0.48	1.00
Cloaca	Surface soil	Brood patch skin	Surface soil	0.09 (0.016)	5.67	**< 0.001**	0.13 (0.017)	8.02	**< 0.001**
Cloaca	Brood patch skin	Feathers	Nest lining	0.04 (0.014)	2.90	0.42	0.05 (0.017)	3.11	0.27
Cloaca	Brood patch skin	Feathers	Surface soil	− 0.01 (0.015)	− 0.83	1.00	0.03 (0.016)	1.60	1.00
Cloaca	Feathers	Feathers	Nest lining	0.10 (0.014)	6.96	**< 0.001**	0.10 (0.017)	5.61	**< 0.001**
Cloaca	Feathers	Feathers	Surface soil	0.04 (0.015)	2.81	0.50	0.07 (0.016)	4.31	**0.005**
Cloaca	Nest lining	Feathers	Nest lining	0.21 (0.014)	15.41	**< 0.001**	0.19 (0.019)	10.08	**< 0.001**
Cloaca	Nest lining	Feathers	Surface soil	0.16 (0.015)	10.71	**< 0.001**	0.16 (0.018)	9.16	**< 0.001**
Cloaca	Surface soil	Feathers	Nest lining	0.14 (0.015)	9.25	**< 0.001**	0.12 (0.018)	6.46	**< 0.001**
Cloaca	Surface soil	Feathers	Surface soil	0.08 (0.016)	5.25	**< 0.001**	0.09 (0.017)	5.25	**< 0.001**
Cloaca	Brood patch skin	Nest lining	Surface soil	− 0.10 (0.015)	− 6.66	**< 0.001**	− 0.09 (0.019)	− 4.92	**< 0.001**
Cloaca	Feathers	Nest lining	Surface soil	− 0.05 (0.015)	− 3.25	0.19	− 0.05 (0.019)	− 2.58	0.72
Cloaca	Nest lining	Nest lining	Surface soil	0.07 (0.015)	4.65	**< 0.001**	0.05 (0.020)	2.30	0.99
Cloaca	Surface soil	Nest lining	Surface soil	− 0.01 (0.016)	− 0.44	1.00	− 0.03 (0.019)	− 1.51	1.00
Brood patch skin	Feathers	Brood patch skin	Nest lining	− 0.16 (0.015)	− 11.15	**< 0.001**	− 0.10 (0.018)	− 5.75	**< 0.001**
Brood patch skin	Feathers	Feathers	Nest lining	− 0.01 (0.014)	− 1.03	1.00	0.00 (0.018)	0.26	1.00
Brood patch skin	Feathers	Brood patch skin	Surface soil	− 0.06 (0.016)	− 3.72	**0.046**	0.02 (0.017)	1.37	1.00
Brood patch skin	Feathers	Feathers	Surface soil	− 0.07 (0.015)	− 4.59	**0.002**	− 0.02 (0.017)	− 1.42	1.00
Brood patch skin	Feathers	Nest lining	Surface soil	− 0.16 (0.015)	− 10.53	**< 0.001**	− 0.14 (0.019)	− 7.37	**< 0.001**
Brood patch skin	Nest lining	Feathers	Nest lining	0.15 (0.014)	10.58	**< 0.001**	0.11 (0.020)	5.44	**< 0.001**
Brood patch skin	Nest lining	Brood patch skin	Surface soil	0.10 (0.016)	6.68	**< 0.001**	0.13 (0.019)	6.74	**< 0.001**
Brood patch skin	Nest lining	Feathers	Surface soil	0.09 (0.015)	6.30	**< 0.001**	0.08 (0.019)	4.25	0.006
Brood patch skin	Nest lining	Nest lining	Surface soil	0.01 (0.015)	0.36	1.00	− 0.04 (0.021)	− 1.81	1.00
Brood patch skin	Surface soil	Feathers	Nest lining	0.04 (0.015)	2.90	0.42	− 0.02 (0.019)	− 0.97	1.00
Brood patch skin	Surface soil	Feathers	Surface soil	− 0.01 (0.016)	− 0.65	1.00	− 0.05 (0.017)	− 2.66	0.64
Brood patch skin	Surface soil	Nest lining	Surface soil	− 0.10 (0.016)	− 6.20	**< 0.001**	− 0.16 (0.020)	− 8.26	**< 0.001**
Feathers	Nest lining	Feathers	Surface soil	− 0.05 (0.014)	− 3.77	**0.040**	− 0.03 (0.019)	− 1.52	1.00
Feathers	Nest lining	Nest lining	Surface soil	− 0.14 (0.014)	− 9.94	**< 0.001**	− 0.15 (0.021)	− 6.97	**< 0.001**
Feathers	Surface soil	Nest lining	Surface soil	− 0.09 (0.015)	− 5.80	**< 0.001**	− 0.12 (0.020)	− 5.92	**< 0.001**

^a^Significant mean differences denoted in bold at *q* < 0.1

### Phylogenetic clustering in bird- and nest-associated bacterial communities

**Fig. 6:**
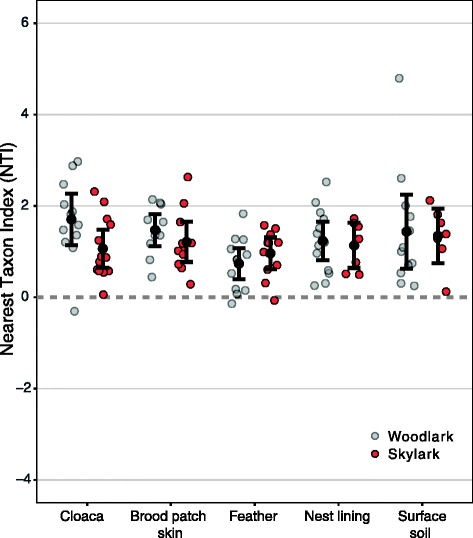
Mean NTI of microbiota of sympatric woodlark and skylarks. The nearest taxon index (NTI) describes the standardised effect size of the observed mean distance to the nearest taxon for all taxa in a community compared to a null distribution. NTI is calculated for each sample and is depicted per sample type and for each lark species separately. Mean NTI values that are significantly different from zero (alpha = 0.05) characterise non-random phylogenetic structure where negative values denote significant phylogenetic overdispersion and positive values denote phylogenetic clustering of bacterial OTUs at the tips of the phylogenetic tree. Sample type means (black circles) and 95% confidence intervals (whiskers) are shown per group

Analysis of mean NTI values for each sample type (i.e. local community) and lark species separately revealed significant non-random phylogenetic structure at the tips of the phylogenetic trees of each sample type (Fig. 6). All sample types were phylogenetically clustered (lower 95% confidence limit > 0). This implies that the taxa found in each sample type were phylogenetically more related than expected in a neutrally assembled community from the same species pool. The mean NTI values did not differ among sample types (*F*_4,100_ = 2.27, *P* = 0.07) or host species (*F*_1,100_ = 1.54, *P* = 0.22). Analysis of mean phylogenetic distances between each pair of taxa, measured as the mean NRI value per sample type, showed a significant deviation from the null distribution in cloacal communities of woodlarks, but not in any other sample type, implying that most of the analysed microbial communities were randomly structured deeper in each sample type’s phylogeny (Figure S7 [Additional file 1]).

## Discussion

Characterising and comparing the microbiotas of sympatric woodlarks *Lullula arborea* and skylarks *Alauda arvensis*, and their nests, we found that the bird-associated microbiotas resembled the environmental microbial communities and concluded that lark-associated microbiota were shaped more by horizontal acquisition than by habitat filtering or host-microbiota coevolutionary history. Patterns of OTU richness, Shannon diversity, dominant taxonomic groups (Proteobacteria, Firmicutes, Actinobacteria, Bacteroidetes and Acidobacteria) and their relative abundances in cloacal, skin and feather communities did not differ between woodlarks and skylarks. Also, ordination analysis of microbiota composition did not separate woodlarks and skylarks in any sample type. Variation in OTU richness and community composition in the three types of bird-associated microbiota (cloaca, brood patch skin and feathers) was partly explained by significant among-individual differences. This was not the case for nest microbiota (nest lining material and surface soil). In addition, the three bird-associated microbiotas harboured few unique and many shared OTUs, of which many were also shared with the nest microbiota. However, using ordination analysis, which also takes relative abundances and phylogenetic relationships into account, we found that samples clustered by sample type, while female identity also had a significant effect. Confirming the effect of individual females, the within-sample type dispersions tended to be higher for the three bird-associated microbiotas than for the nest-associated communities. In all sample types, patterns of phylogenetic community structure revealed significant but weak clustering at the OTU level, not at taxonomic levels deeper in the phylogeny. Here, we first compare our lark microbiota characteristics with microbiota of other birds. We then discuss the implications of our findings at the levels of host species, individual and body part for the evolutionary and ecological factors that shape variation in host-microbe associations. Finally, we discuss the community assembly processes that may govern bird microbiota assembly.

### Microbiota of woodlarks and skylarks resemble other avian microbiota

The cloacal microbiotas of woodlark and skylark resembled those of other (passerine) bird species with respect to Shannon diversity [76] and the dominant bacterial groups [76–80]. Unfortunately, we cannot compare our OTU richness estimates with other studies, because OTU binning and sequencing/rarefaction depth strongly determine OTU richness estimation. Because our study is the first to describe the avian brood patch skin and because feather microbiotas of wild birds have not been previously characterised based on sequencing data, we cannot compare the results of these body parts to other species. Nevertheless, we showed that the dominant bacterial phyla (Proteobacteria, Actinobacteria, Firmicutes, Bacteroidetes and Acidobacteria) were the same in cloaca, brood patch skin and feathers. These bacterial phyla, with the exception of Acidobacteria, have also been found to dominate the cloacal microbiota of other studied passerines [77, 79] and non-passerines [78]. A potential explanation for the dominant presence of Acidobacteria in cloacal, brood patch skin and feather microbiota of our species is their also dominant occurrence in the larks’ nest microbiota.

### Interspecific comparison: a large role for the environment in shaping bird microbiota

The small differences between woodlark and skylark in alpha diversity, dominant bacterial taxa and community composition of the microbiota of cloaca, brood patch skin and feathers collectively suggest that the shared environment/ecology is more important than the different host evolutionary histories in shaping these microbiota. Our findings do not support the phylosymbiosis hypothesis [26], which postulates that microbiota are host-specific as a result of coevolutionary history between host and microbiota. Phylosymbiosis is supported by studies on passerine birds [80] and other taxa [26, 29] or partially supported by studies on birds [77] and mammals [81] that demonstrate simultaneous effects of ecology and phylogeny on host-microbiota. However, our lark findings are in line with a series of investigations on birds, mammals and reptiles that also do not find support for phylosymbiosis and instead demonstrate a lack of interspecific microbiota differences among sympatric species [82] or strong microbiota convergence due to sympatry [15] and dietary similarity [31, 33]. To cover these studies, we propose the ‘niche-driven microbiota assembly hypothesis’ as an alternative to the phylosymbiosis-hypothesis, stating that host-microbiota associations can be shaped by environmental and/or ecological factors instead of coevolutionary history.

### Bird-associated microbiota vary among individuals

Differences among individual females explained 18% of the richness, and 20% of the community composition based on the three bird-associated sample types, but had no explanatory power for the Shannon diversity. Variation in nest-associated sample types was not explained by individual for either Shannon diversity, richness or composition. The inter-individual variation in bird-associated microbiota raises the questions whether they are maintained consistently over time and whether they are caused by genetic or environmental effects. To determine whether differences in host-associated microbiota among individuals are consistently maintained over time requires longitudinal sampling [83]. Studies in free-living animals thus far show mixed results: Microbiota of chimpanzees *Pan troglodytes* monitored over 8 years [84] and of barn swallows *Hirundo rustica* followed during a breeding season [85] showed individual consistency, while microbiota of deer mice *Peromyscus spp.* were not repeatable over merely 1 week [82]. Studies determining whether inter-individual differences in host-associated microbiota can be attributed to genetic or environmental effects mainly contributed individual variation to environmental effects [76, 86, 87], supporting our niche-driven microbiota assembly hypothesis.

### Resemblance of microbiota among body parts and nest environment indicates horizontal transmission

OTU co-occurrence and community resemblance patterns between bird-associated and nest-associated microbiota showed overlap among body parts and with nest samples, suggesting only weak habitat filtering at the level of the body part. In humans, the microbes on the body demonstrated great overlap with indoor-environment microbiota [88, 89], but in terrestrial vertebrates, only one study has simultaneously measured and compared environmental and animal microbiota [90]. This study, on wild American redstarts *Setophaga ruticilla*, compared microbial communities on feathers and in soil and found that they significantly differed, suggesting that soil plays a minor role in shaping plumage microbiota [90]. This finding is opposite to our lark results, which may be due to ecological differences between the species: redstarts are arboreal foragers and larks are ground foragers. In addition, the redstart diversity values, measured using length heterogeneity PCR, were low and probably underestimated as compared to present-day Illumina sequencing results [91] applied in our lark study. Mechanisms that might foster transfer between environment and animal include diet [34, 36], direct contact with environmental sources such as soil microbiota in ground foragers [92], or interindividual contact [79, 85].

Our finding in both lark species that the microbiota of different body parts (cloaca, brood patch skin, feathers) resembled each other was in contrast with the single other bird study using next-generation sequencing data that compared microbiota among body parts, namely hindgut and facial skin from carcass-eating vultures, and that reported no overlap [42]. A study of a murine model showed more overlap in composition between lung and vaginal microbiota, than each overlapped with caecal microbiota [93], and resemblance among body parts in humans also indicated that differences in habitat filtering and/or varying degrees of horizontal uptake shaped the microbiota of different body parts [13], corroborating our findings. The lower diversity and potentially reduced richness in the larks’ cloacal microbiota compared with their skin, feather and nest communities may result from more intensive top-down regulation by host genetic factors [94] or immune function [95] in the intestine/cloaca. Brood patch skin microbiota most strongly resembled feathers and soil, suggesting that horizontal uptake from the surrounding microbiota was profound. The microbiota on feathers were richer than cloacal, brood patch skin and soils and shared a majority of OTUs with all bird-associated and nest-associated microbiota, suggesting that feathers also horizontally acquired bacterial symbionts from multiple sources. Because OTU richness but not Shannon diversity of feather microbiota exceeded that of other bird-microbiota, we suggest that many taxa on feathers may only be present as low-abundant transient members, which could be expected from horizontal acquisition. For ground-foraging larks, it may not be surprising that feathers acquire bacteria from the soil, but the resemblance among these communities also emphasises that habitat filtering is weak in feathers. Collectively, we conclude that microbiota of different body parts horizontally acquire microbes from each other and from environmental communities, consistent with our niche-driven microbiota assembly hypothesis.

### Phylogenetic community structure in bird-associated samples

Given significant but weak phylogenetic clustering at the OTU level (NTI) of cloacal, brood patch skin and feather microbiota, we concluded that habitat filtering [70, 96] plays a role in shaping the bird-associated microbiota of our larks. These results corroborated our findings that the different bird-associated sample types (cloaca, brood patch skin, feather) differed in composition (Fig. 4). We did not observe phylogenetic clustering deeper in the phylogenies (NRI) of any of the bird-associated microbiota. However, because our phylogenetic tree was based on the conserved 16S rRNA gene and comprised many (1148) OTUs with much expected functional redundancy [13, 96], we caution that in our study, NRI analyses cannot be interpreted as absence of habitat filtering at higher taxonomic levels. The fact that the phylogenetic clustering at the OTU level was relatively weak compared with NTI values in other bacterial communities [97, 98] suggests that there was no strong filtering within the microbial communities present in/on the various body parts. Together with high levels of OTU co-occurrences and strong compositional resemblance among sample types, this weak phylogenetic clustering of bird-associated microbiota provides scope for acquisition of OTUs from the bird’s environment onto the bird’s body, which would be a prerequisite for our niche-driven microbiota assembly hypothesis.

## Conclusion

The sympatric occurrence of two lark species (Alaudidae) enabled us to test, by interspecific comparison of breeding females, if host evolutionary history would generate microbiota differences, while sharing breeding habitat and other resources. Our data showed that the cloacal, skin and feather microbiota did not differ in alpha diversity, community composition and phylogenetic community structure between woodlarks and skylarks. Based on comparisons of the composition and dominant bacterial taxa of bird- and nest-associated microbiota, we observed associations among the various body sites and with the nest environment. Patterns of phylogenetic structure of cloacal, skin and feather microbiota suggested weak filtering at each niche. All these patterns were consistent between both lark species, and we therefore suggest that a shared (spatial) environment, and shared ecological factors (e.g. diet), may have avoided these species’ microbiotas to differ. These observations raise the hypothesis that sharing an ecological niche among hosts (either species or individuals) leads to convergence of their microbiota. Comparative microbiota studies are typically challenged by the confounding nature of ecological and phylogenetic divergences among hosts, which hampers their use to discern phylogenetic from ecological driving factors. In order to discern evolutionary and ecological effects on interspecific microbiota variation, based on this study, we believe that it is important in future studies either to compare species inhabiting a similar ecological niche to test for effects of host evolutionary history or to limit the phylogenetic breadth of host species to test ecological factors.

## Additional files

Additional file 1: Supplementary information. (DOCX 170096 kb)

Additional file 2: Metadata file for creation of phyloseq object. (TXT 14 kb)

Additional file 3: Pipeline for sequence data processing. (TXT 18 kb)

Additional file 4: OTU community table file for creation of phyloseq object. (BIOM 1676 kb)

Additional file 5: Phylogenetic tree file for creation of phyloseq object. (TRE 7216 kb)

Additional file 6: Preprocessed, quality filtered raw sequence data file. (FASTA 74532 kb)

Additional file 7: R script file for statistical analysis. (R 77 kb)
