# Supplementary material for: Multi-level comparisons of cloacal, skin, feather and nest-associated microbiota suggest considerable influence of horizontal acquisition on the microbiota assembly of sympatric woodlarks and skylarks
Source: Microbiome. 2017 Dec 1;5:156. doi: 10.1186/s40168-017-0371-6 (PMC5709917; doi:10.1186/s40168-017-0371-6)
Supplement: Supplementary file 1 — Supplementary information. (DOCX 170096 kb) [file 40168_2017_371_MOESM1_ESM.docx]

**Additional file 1**

**Supplementary figures and tables**

**Figure S1.** Rarefaction curves of samples from different origins. a) Rarefaction curves depict the estimated average a) OTU richness (97% ID), b) Shannon diversity and c) Chao1 (whiskers represent ± 2 SD) which are based on 10 samplings for each rarefaction depth. Samples are coloured by sample type of origin. Despite the maximum OTU richness and predicted total diversity (Chao1) have not reached a plateau at rarefaction depth 5000, Shannon diversity levelled off. Although the current sequencing efforts were unable to capture the total diversity, Chao1 values (c) show some separation of total diversity estimates for different sample types.

**Figure S2.** Rank-abundance plots for each sample type. Dotplots depict rank-abundance plots for the 500 most abundant phylotypes of each sample type. Note that the y-axes are log10 transformed. The patterns are highly skewed with few very abundant and many rare phylotypes.

**Figure S3.** Bacterial community characteristics with an OTU table constructed on all high-quality reads. a) Boxplots depict phylotype richness (97% ID) and b) Shannon diversity of sample types associated with woodlarks (grey) and skylarks (red). c) Principal coordinate (PCoA) plot of weighted UniFrac shows the bacterial community composition of all samples, coloured by sample type for woodlarks (circles) and skylarks (triangles). a, b) Letters denote group differences determined by Dunn’s test for multiple comparisons with FDR q < 0.05. Axis 1 and 3 are shown for most intuitive cluster visualisation. Group dispersions differ between sample types (betadisper: *F*_4,104_ = 5.60, *P* < 0.01), and thus warrants cautious interpretation of statistical support for differential clustering. Sample type explains about 30% of variation in weighted UniFrac.

**Figure S4.** Barplots of the most dominant OTUs in body site-specific bird-associated microbiota. OTUs are included when the mean relative abundance of an OTU exceeded 5% across all samples of a sample type, separately evaluated for each lark species. Relative abundances of dominant OTUs are depicted for (a, b) cloacal, (c, d) brood patch skin and (e, f) feathers from woodlarks and skylarks, respectively. OTU bars are split by bacterial phylum and coloured by family. Dominant OTUs belong to the same phyla in each body site of both lark species, but the identity (and family) of the dominant OTU varies in the Actinobacteria and Firmicutes in cloacae and skin communities, and in Bacteroidetes in feather communities. Bars represent individual samples that are labelled along the x-axis by sample ID.

**Figure S5.** Beta diversity of bacterial communities associated with different sample types of sympatric lark species. Principal coordinate analysis on Bray-Curtis dissimilarities between sample types is shown along the first three principal coordinate axes and was calculated on rarefied data (5000 reads/sample). Sample types significantly clustered, explaining 26% of variation (PerMANOVA, pseudo-*F* = 10.1, df = 4, 104, *R^2^ =* 0.26, *P* < 0.001) and woodlarks and skylarks only weakly clustering explaining 1% of variation (pseudo-*F* = 2.13, df = 1, 104, *R^2^* = 0.01, *P* < 0.05). Female ID explained 23% of total variation in community clustering (PerMANOVA, pseudo-*F* = 1.26, df = 27, 77, *R^2^* = 0. 22, *P* < 0.01).

**Figure S6.** Clustering of samples through partitioning around medoids. a) Lineplot shows gap statistic of K clusters as identified using partitioning around medoids (PAM). b) NMDS ordination plot shows samples coloured by K clusters at K=5. c) NMDS ordination plot shows samples clustered by K clusters at K=5 and coloured by sample type for delineated by host species (symbol shape). Stress in both NMDS plots is 0.14.

**Figure S7.** Mean NRI across sample types of sympatric lark species and their nest environment. The Net Relatedness Index (NRI) describes the standardised effect size of the observed mean phylogenetic distance among every pair of taxa in a community compared to a null distribution. NRI is calculated for each sample and is depicted per sample type and for each lark species separately. Mean NRI values that are significantly different from zero characterise non-random phylogenetic structure at deep branching of the phylogenetic tree (as opposed to NTI) where negative values denote significant phylogenetic evenness and positive values denote phylogenetic clustering of bacterial lineages. Sample type means (black circles) and 95% confidence intervals (whiskers) are shown separately for both woodlarks and skylarks for each sample type.

| **Woodlark** | | | | |
| --- | --- | --- | --- | --- |
|  | | n collected | n sequenced | Remarks |
| Cloacal gut | | 14 | 12 | V563807 and V758029 failed PCR amplification |
| Brood patch skin | | 13 | 11 | V637773 failed PCR and V573280 removed due to low coverage (1049 reads) |
| Feathers | | 13 | 13 |  |
| Nest lining | | 14 | 13 | Nest B14.140 failed PCR amplification |
| Surface soil | | 14 | 12 | Nest B14.11 and B14.54 contained high humic acid content in DNA extract causing PCR amplification failure |
| **Skylark** | | | | |
| Cloacal gut^a^ | 14 | | 13 | V563807 failed PCR amplification |
| Brood patch skin | 11 | | 11 |  |
| Feathers | 11 | | 11 |  |
| Nest lining^b^ | 7 | | 7 |  |
| Surface soil^b^ | 9 | | 7 | Nest V14.16 and V14.42 failed PCR amplification |
| Total count | 120^c^ | | 110 |  |

**Supplementary Table S1.** Overview of collected and successfully sequences samples.

a	Females captured for another study were only sampled for cloacal microbiota assessment.

b	Nest material and soil samples were not collected for females captured away from their nests, or at replacement nests found	after female sampling.

c	Complete sets of samples for all females (n = 29) would yield 145 samples, but nest failure constrained sample collection. A complete list of sample metadata is provided in the mapping file [Additional file 5].
